# Targeted delivery of organic small-molecule photothermal materials with engineered extracellular vesicles for imaging-guided tumor photothermal therapy

**DOI:** 10.1186/s12951-023-02133-5

**Published:** 2023-11-23

**Authors:** Yafang Dong, Peng Xia, Xiaolong Xu, Jing Shen, Youbin Ding, Yuke Jiang, Huifang Wang, Xin Xie, Xiaodong Zhang, Weihua Li, Zhijie Li, Jigang Wang, Shan-Chao Zhao

**Affiliations:** 1 Department of Urology, the Third Affiliated Hospital of Southern Medical University, Guangzhou, Guangdong 510500 P. R. China; 2 Department of Nephrology, Shenzhen Key Laboratory of Kidney Diseases, Shenzhen Clinical Research Centre for Geriatrics, Shenzhen People’s Hospital, The First Affiliated Hospital, Southern University of Science and Technology, Shenzhen, Guangdong 518020 P. R. China; 3 Department of Hepatobiliary & Pancreatic Surgery, Zhongnan Hospital of Wuhan University, Wuhan, Hubei 430072 P. R. China; 4 Department of Oncology, Department of Infectious Disease, Shenzhen People’s Hospital, The First Affiliated Hospital, Southern University of Science and Technology, Shenzhen, Guangdong 518020 P. R. China; 5 Department of Medical Imaging, The Third Affiliated Hospital of Southern Medical University, Guangzhou, Guangdong 510630 P. R. China; 6 Medical imaging department, Shenzhen Second People’s Hospital/the First Affiliated Hospital, Shenzhen University Health Science Center, Shenzhen, Guangdong 518035 P. R. China; 7 State Key Laboratory for Quality Ensurance and Sustainable Use of Dao-di Herbs, Artemisinin Research Center, Institute of Chinese Materia Medica, China Academy of Chinese Medical Sciences, Beijing, 100700 P. R. China; 8 Department of Oncology, the Affiliated Hospital of Southwest Medical University, Luzhou, Sichuan P. R. China; 9 Department of Urology, Nanfang Hospital, Southern Medical University, Guangzhou, Guangdong 510515 P. R. China

**Keywords:** Imaging-guided photothermal therapy, Small organic photothermal agents, Croconaine dyes, Extracellular vesicles, Active tumor targeting

## Abstract

Imaging-guided photothermal therapy (PTT) for cancers recently gathered increasing focus thanks to its precise diagnosis and potent therapeutic effectiveness. Croconaine (CR) dyes demonstrate potential in expanding utility for near infrared (NIR) dyes in bio-imaging/theranostics. However, reports on CR dyes for PTT are scarce most likely due to the short of the efficacious delivery strategies to achieve specific accumulation in diseased tissues to induce PTT. Extracellular vesicles (EVs) are multifunctional nanoparticle systems that function as safe platform for disease theragnostics, which provide potential benefits in extensive biomedical applications. Here, we developed a novel delivery system for photothermal molecules based on a CR dye that exerts photothermal activity through CDH17 nanobody-engineered EVs. The formed CR@E8-EVs showed strong NIR absorption, excellent photothermal performance, good biological compatibility and superb active tumor-targeting capability. The CR@E8-EVs can not only visualize and feature the tumors through CR intrinsic property as a photoacoustic imaging (PAI) agent, but also effectively retard the tumor growth under laser irradiation to perform PTT. It is expected that the engineered EVs will become a novel delivery vehicle of small organic photothermal agents (SOPTAs) in future clinical PTT applications.

**Supplementary Information:**

The online version contains supplementary material available at 10.1186/s12951-023-02133-5.

## Introduction

Cancer is a main driver of human mortalities globally [1]. It is urgently needed to develop methods for precise diagnosis of cancers, especially early diagnosis, improve treatment for primary and metastatic cancers, and reduce mortality [2]. Currently, imaging-guided therapy has attracted great attention for its high efficiency, accurate and precise localization for irradiation treatment and minimal invasiveness to normal tissues. Photoacoustic imaging (PAI) is capable to visually monitor organs or tissues in multidimension, showing increased sensitivity together with high spatial resolution, intense penetration, and good biocompatibility [3]. PAI has been extensively utilized in tumor diagnosis, drug biodistribution and metabolism [4], and imaging-guided surgery and treatment [5].

Photothermal therapy (PTT) reflects a promising and highly effective treatment option for various diseases including cancers [6–8]. PTT takes advantage of localized heat derived from photothermal agents (PTAs) exposed to near-infrared (NIR) irradiation to realize tumor elimination [9]. Meanwhile, PTT has been combined with other therapeutic modalities, such as chemodynamic therapy (CDT) and photodynamic therapy (PDT), to boost the anti-tumor effect or prevent multidrug resistance [8]. The PTT efficacy is mainly determined by the photothermal conversion efficiency (PCE) together with biocompatibility for PTAs [10]. PTAs with high PCE and good biocompatibility will largely improve the performance for tumor therapy. Three types of PTAs, including inorganic nanomaterials, organic small molecular dye and conjugated polymer nanoparticles (NPs) have been developed to achieve efficient PTT over the last decades [11]. These PTAs accumulated in diseased tissues usually absorb NIR light with the capacity for transferring absorbed light into thermal energetic sources whereas the living cultures nearby the diseased tissues exhibit no absorption and autofluorescence [12]. The clinical translation of inorganic nanomaterials is potentially thwarted owing to their limited biocompatibility, biodegradability and possible extended half-life safety concern. The conjugated polymer-based PTAs typically possess acceptable absorbance profiles within near-infrared II (NIR-II) window due to such well-conjugated frameworks. While the reduced solubility/metabolic features restrict their practical applications [13, 14]. In comparison, small-molecule organic photothermal agents (SOPTAs) show many irreplaceable superiorities for disease treatment due to their structural universality, easily adjusted optical properties, excellent biocompatibility, intrinsic biodegradability and low toxicity [15, 16]. Therefore, SOPTAs have received increasing interest for biomedical applications in recent years. Nevertheless, many of the reported SOPTAs are subject to poor photostability, low PCE and poor tumor accumulation for clinical applications of PTT. Thus, it’s desirable to develop stable SOPTAs with strong NIR absorption for highly efficient PTT, which is engaging but demanding.

Croconaine (CR) dyes are zwitterionic compounds with extended π-conjugation greatly promoted the electron resonance and energy transfer [17, 18]. CRs exhibit sharp and intense NIR absorption [19], and superior chemical/thermal/photo-stability [20]. Moreover, unlike other reported organic small molecule dyes, such as cyanine, polymethine, squaraine, benzo[1,2-c:4,5-c′]bis ([1, 2, 5]thiadiazole) (BBTD), 4,4-difluoro-boradiazaindacene (BODIPY), and tetrapyrroles, which normally need complex synthetic routes, CRs can be readily synthesized and chemically modified [21]. Therefore, there exists a need for constructing novel CR dyes for future bio-imaging and theragnostics. Multiple issues, however, beset traditional SOPTAs on their in vivo applications: inherent hydrophobicity, short circulation half-life, rapid clearance, weak tumor targeting ability [16]. Based on nanotechnologies widely used in recent years, the hydrophobic organic small molecules are generally encapsulated into or conjugated with nanomaterials/polymer matrices, such as liposomes, proteins, and metals to form nanoparticles to accomplish their in vivo biological applications [22]. To date, the most of nanoparticle systems accumulate into tumors across passive targeting orchestrated through enhanced permeability and retention (EPR) functions; some PTAs have been endowed with the active targeting ability to tumors through nanocarriers modified with specific ligands targeting tumor sites [6].

EVs are proteolipid bilayer enclosed nanovesicles (30–150 nm) released naturally from all the cultures [23]. They mediate intercellular communication in tumor microenvironment. EVs possess remarkable biocompatibility, innate stability, low toxicity, low immunogenicity, high engineerability and intrinsic ability to cross biological barriers [24, 25]. Thus, EVs are emerging as ideal nanocarriers for targeted delivery of drugs or biomaterials for disease diagnosis and therapy; EVs isolated from various cells, such as normal cells, immune cells and tumor cells, have been explored as drug delivery vectors or therapeutic agents to treat diverse diseases in preclinical settings including cancers [26]. However, due to the nonspecific uptake of EVs in normal tissues, the unwanted side effects could occur [27]. Therefore, how to turn EVs specifically accessing their targets is the main challenge for EVs applications. To this end, methods for surface modification of EVs with specific targeting ligands to enhance their delivery specificity have been extensively studied. Up to now, many studies described the conjugation of EVs with specific peptides, antibodies or antibody fragments and nanobodies to achieve the targeting ability for diagnosis and treatment in a range of diseases [28].

**Scheme 1:**
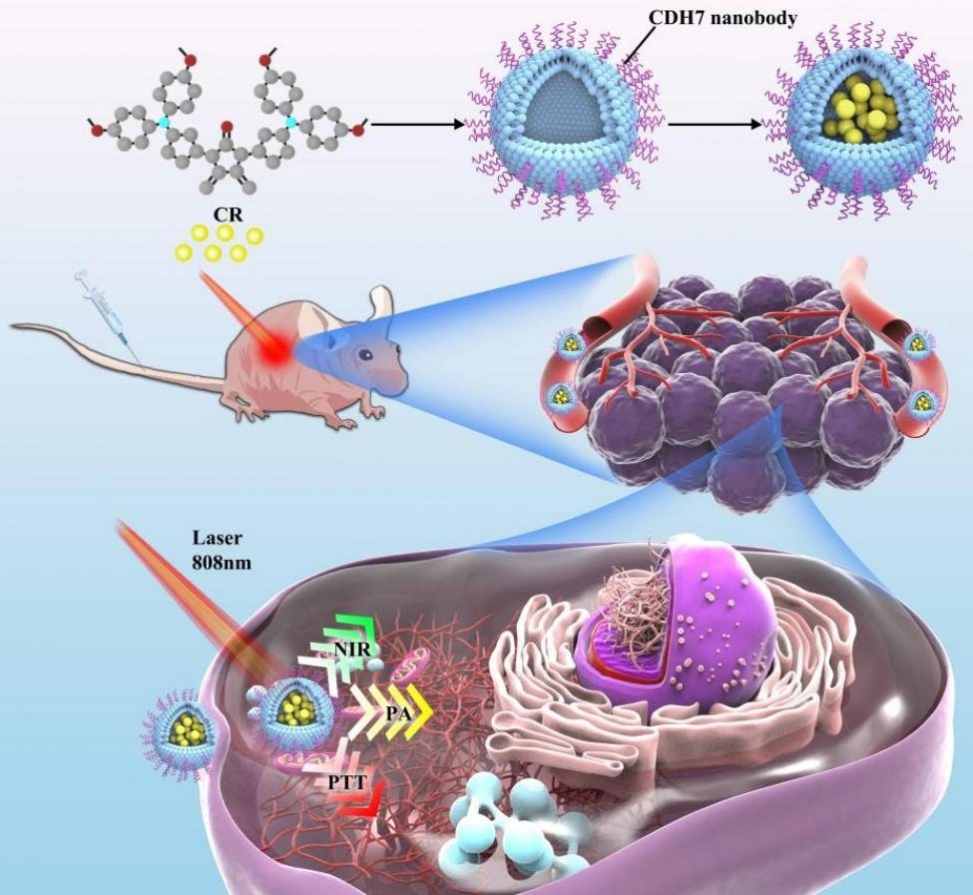
Graphical depiction for specific delivery of CR dyes with nanobody engineered EVs for tumor targeting PTT guided by PAI.

Cadherin 17 (CDH17) is a calcium-dependent cell adhesion glycoprotein involved in tumor invasion and metastasis [29]. Studies have shown that CDH17 is a cell surface biomarker highly expressed in gastrointestinal (GI) cancers, such as colorectal, gastric and pancreatic cancers, while it only shows a restricted expression in normal tissues [30–32]. Therefore, CDH17 is an ideal target for GI cancer diagnosis and treatment. Two nanobodies (A1 and E8) having good affinity and specificity against to CDH17 protein were identified and characterized by our lab [33], one of the previously work has also utilized the CDH17 nanobody engineered EVs for imaging.

of gastric cancer and efficient drug delivery [34]. Here, we firstly reported that CDH17 nanobody E8 engineered EVs secreted from normal human embryonic kidney (HEK-293) cultures were loaded with a NIR SOPTA (CR-DPA-T) for PA imaging-guided PTT in a gastric cancer model (Scheme 1). The simple modification led to the formation of CR@E8-EVs with the enhanced functional strength for both CRs and EVs, including high photostability, rapid distribution/renal clearance together with minimized aggregation within healthy tissue, active tumor-targeting/high tumor accumulation and retention. We demonstrated that CR@E8-EVs could specifically target the CDH17-positive gastric cancer cells, and accomplish the PA imaging-guided PTT in a gastric tumor model leveraging excellent photothermal conversion capability of CR-DPA-T. Notably, only a single-dose administration of CR@E8-EVs and once 808 nm laser irradiation achieved excellent photothermal ablation of tumors. This study builds an efficient targeting nanoplatform for tumor therapy based on croconaine dyes and engineered EVs, which might speed up the clinical translation of small organic molecular dyes for biomedical application.

## Methodology

### Cellular culturing

MKN45 and 4T1 cells were grown in RPMI1640 containing 10% fetal bovine serum (FBS) plus 1% antibiotics. HEK-293 and IM95 were grown within DMEM with 10% FBS plus 1% antibiotics. Cells were grown in a incubator with 37 °C/5% CO_2_.

### EVs isolation

EVs isolation was performed based on the previous study from genetic engineered HEK-293 cultures [28]. In brief, cultures were kept within exosome-free FBS (72 h) for product EVs. The cell-free conditioned media were exposed to centrifuging (500 g/4°C/10 minutes; 12,000 g/20 minutes; 20,000 g/30 minutes) for removing non-living cultures/cellular debris. Subsequently, media were filtered through a 0.22 μm filter and then ultracentrifuged at 200,000 g for 120 min. Finally, EVs pellets were re-suspended through Trehalose PBS and placed into storage (− 80 °C) until use. NTA was conducted through a NanoSight® NS300 system and size distribution was determined through Stokes − Einstein equation. Morphological features for isolated EVs were acquired through transmission electron microscopy (TEM).

### Preparation of CR@E8-EVs

E8-EVs (5 × 10^9^ mL^− 1^ in PBS, 0.5 mL) and CR-DPA-T (1 mg mL^− 1^ in water, 0.5 mL) were suspended in a 1.5 mL of centrifugal tube. The mixture was then transformed into an 4 mm electroporation cuvette for electroporation (Bio-Rad), and treated with 200 V, 1 ms. The obtained solution was again transported into a 1.5 mL centrifugal tube, consequently being incubated (4 °C/overnight).

### Photo-physical profiling

UV-Vis-NIR absorption spectra were determined at RT (room temperature) upon 201 UV-visible spectrophotometers (Thermal Fisher Scientific, USA) with 1 nm resolution, through quartz cuvettes (1 cm path-length).

### Photothermal function / PCE determinations for CR@E8-EVs

The temperature response of the sample was evaluated by the 808 nm laser (Shenzhen Purui Material Technology Co., Ltd., Shenzhen, China). Meanwhile, sample temperature was recorded through IR thermal imaging.

To investigate the photothermal performance of CR@E8-EVs, 50 µg/mL CR@E8-EVs under irradiation with 808 nm laser at various power intensities (0.1, 0.25, 0.5, 0.8, and 1.0 W/cm^2^) were examined. Moreover, the temperature changes across differing doses (10, 25, 50, and 100 µg mL^− 1^) of CR@E8-EVs upon exposure to the laser (0.8 W/cm^2^, 360 s) were also recorded. PBS was used as the control. To explore photothermal stability, sample temperature was determined across five heating − cooling cycles. Within each cycle, NIR laser was initially irradiated samples (five minute-period) for obtaining steady-state. Once laser was deactivated, samples were left to cool down for five minutes. The photothermal conversion efficiency (PTCE, ƞ) for CR@E8-EVs was then determined at 72% according to the reference and obtained data (Fig. S3) [18].

### Cytotoxicity assessment

CR@E8-EV cytotoxicity analysis was performed on MKN45 cells using CCK-8 viability assessment. Briefly, cells were seeded in 96-well plates (1 × 10^4^ cells/well; 24 h incubation). Then cells were cultured with 0–50 µg/mL CR@E8-EVs for 4 h. After incubation, cultures were handled with/without of laser irradiation (808 nm, 0.8 W/cm^2^) for five minutes. Post-12-hour-incubating, supernatant was changed using PBS (90 µL)/CCK-8 (10 µL), with subsequent four-hour-incubating period and final analyses through microplate reading (450 nm).

### Live/dead cell-staining assessment

Live/dead cell-staining against MKN 45 cultures was performed with the calcein-AM/PI solution. MKN45 cells were grown within 6-well plates (1 × 10^5^ cells/well), with a day for acclimatization. Cultures were consequently exposed as described here: PBS, E8-EVs, CR or CR@E8-EVs (25 µg/mL), with/without 808 nm laser irradiation (0.8 W/cm^2^, 5 min). Following a further 12-hour incubation, treated cultures were rinsed thrice through PBS, and consequently stained with calcein-AM (2.0 µM) and PI (4.5 µM) for 30 min within the cell-cultured container. Finally, cultures were PBS-rinsed/exposed to imaging through confocal microscopy.

### Flow cytometry experiments

MKN45 cultures were treated with the same way as above and cultured overnight. Then, the cultures were trypsinized, washed, and stained with Annexin V-FITC/PI-stained (Apoptosis Detection Kit) according to manufacturer’s protocol.

### Animals/tumor-bearing mouse model

All experiments on the animals were performed in line with accepted recommendations of the Institutional Animal Care and Use Committee (IACUC), Shenzhen People’s hospital, and performed under legal protocols. In this study, 6–8 week-old female BALB/c nude mice (≈ 20 g) were used (Gempharma Tech™, Guangzhou, China), and kept in Specific Pathogen Free (SPF) conditions within animal center (Shenzhen People’s hospital). To obtain the xenograft MKN45 tumor-bearing mouse model, the 100 µL PBS suspension containing 1 × 10^6^ MKN45 cells were subcutaneously transplanted within mouse right-back. For 4T1 tumor-bearing mouse model, the 100 µL PBS suspension containing 3 × 10^6^ 4T1 cells were injected. Once tumor dimensions approached 150 mm^3^, the tumor-bearing mice were used for subsequent in vivo imaging/PTT investigations: tumor volume = (tumor length) × (tumor width)^2^/2. Across all in vivo investigations, mice were randomly grouped.

### Targeting ability evaluation of E8-EVs

DIR@control-EVs (100 µL) and DIR@E8-EVs (100 µL, 0.5 mg/mL based on DIR) were intravenously administered within MKN45 tumor-bearing mice, with real-time fluorescent signal changes of mice across specific timepoints (4, 8, 12, and 24 h) being acquired through IVIS imaging. One day later, mice designated for imaging were sacrificed and major organs (heart, liver, spleen, lung, and kidney) and tumor mass were removed for ex vivo imaging. Imaging was analyzed by Image J package.

### In vivo PA imaging of CR@E8-EVs

For in vivo PA imaging, CR@E8-EVs (100 µL, 0.5 mg mL^− 1^, based on CR) were intravenously administered within MKN45 tumor-bearing mice, and free CR (100 µL, 0.5 mg mL^− 1^) was used as the control. PA signals or images were monitored by a commercial Fujifilm VisualSonics Vevo® LAZR-X system at different post-injection timepoints (4、8、12 and 24 h). The averaged tumor tissue PA signals were acquired by Vevo LAB 5.5.1 package.

### In vivo infrared thermography and photothermal therapy

In vivo anti-tumor study of CR@E8-EVs was conducted within MKN45 tumor bearing mice (BALB/c). Thirty female mice were randomly divided into six cohorts, with five mice in each group, once tumor volume approached 150 mm^3^. Such six cohorts were PBS, PBS plus laser, CR, CR plus laser, CR@E8-EVs and CR@E8-EVs plus laser, and subjected to different treatments as follows: injection of 100 µL (1) PBS, (2) PBS plus laser, (3) free CR (0.5 mg mL^− 1^), (4) free CR (0.5 mg mL^− 1^) plus laser, (5) CR@E8-EVs (0.5 mg mL^− 1^ on CR basis), and (6) CR@E8-EVs (0.5 mg mL^− 1^ on CR basis) plus laser. For laser irradiation cohorts (2), (4) and (6), mice were anesthetized through 2% isoflurane in oxygen flow, with tumor areas continuously exposed to irradiation through an 808 nm laser (0.8 W cm^− 2^, 10 min) after twelve hours post-injection. Meanwhile, thermal images/real-time temperature shifts across tumor areas of each cohort were photographed and recorded through infrared thermal imaging. Tumor volumes and body weight of all the mice were determined through Vernier caliper/electronic balance daily to examine therapeutic capacity for CR@E8-EVs during the whole study after various treatments. Drug and irradiation therapies were solely administered once.

### Histopathological analysis

All mice were dissected post-treatment, the tumors / major organs were collected for further biosafety evaluation of materials and the efficacy of PTT. H&E staining was performed for main organs mainly including heart, liver, spleen, lung, and kidney. Fluorescent TUNEL staining was performed according to kit protocol for DeadEnd® fluorometric TUNEL system kit (Promega,™, USA). Apoptotic cells were viewed through a microscope. Regarding hematoxylin and eosin (H&E) staining, tissue samples were 4% formalin-fixed, paraffin-embedded, followed by Sect. (5 μm girth). Staining was analyzed through digital microscopy.

### Statistical analyses

All the quantitative datasets reflected mean ± SEM unless otherwise specified. Statistical analyses were conducted through GraphPad Prism 8.3.1 package. Two-sided student’s t-test was assessed two-cohort variations. *p* value < 0.05 was deemed to confer statistical significance.

## Results/discussion

### CR-DPA-T synthesis and characterization

**Scheme 2:**
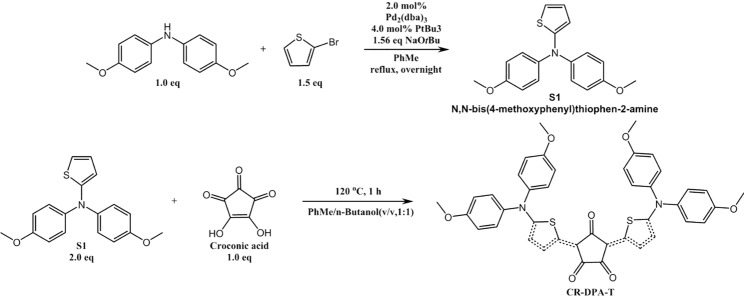
Synthetic route for CR-DPA-T

D-A-D type CR-DPA-T was synthesized through a facile two-step reaction according to previously reported method with commercially available materials, as shown in Scheme 2 [35]. Condensation of electron-withdrawing croconic acid (A) with two equivalents of two strong electron-donating diphenylamine (DPA) units linked to thiophene linkers at 1:1 azeotropic mixture of benzene and n-butanol at 110 ^o^ C, yielding CR-DPA-T as a black solid (28%). All spectral data of the intermediates (S1) and CR-DPA-T matched well with that of previous report [34]. Meanwhile, the D–π–A–π–D CR-DPA-T framework led toward considerable electron delocalization and red-shifted absorption, resulting in excellent high chemical/thermal/photo stability [36, 37].

### Characterization of EVs engineered with CDH17 nanobody

**Fig. 1:**
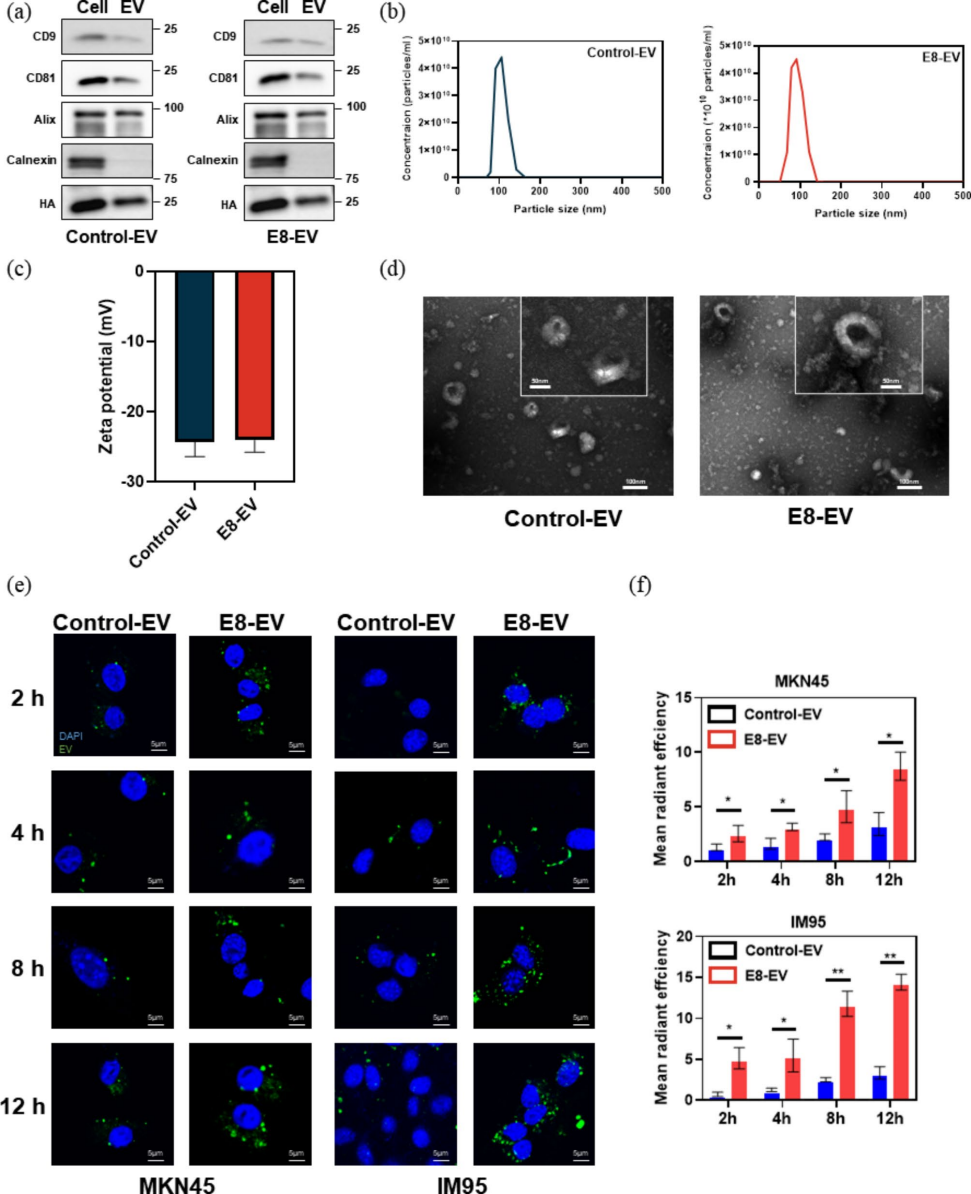
**(a)** Western blot analysis of specific EV biomarkers and HA tag. **(b)** NTA for EVs. **(c)** The zeta potentials for EVs. **(d)** TEM image for EVs. Inset is the magnified image of EVs. Scale bars, 100 nm and 50 nm respectively. **(e)** The internalization efficiency of EVs labeled with PKH67 (green) in the CDH17-positive MKN45 and IM95 cells under different time points. Scale bars, 5 μm. **(f)** The quantification of internalization of EVs for e. Data are expressed as mean ± SEM

It was reported that CDH17 is an ideal diagnostic marker for GI tract cancers [29–34]. Our previous work had confirmed the targeting and cell uptake ability of EVs engineered with CDH17 nanobody E8 [34]. Here, we recapitulated the characteristics of E8-EVs and control nanobody-EVs (Con-EVs) using specific EVs positive biomarkers (CD9, CD81, Alix), together with negative biomarker (Calnexin) (Fig. 1a). HA tag was detectable on the EVs, indicating the nanobodies fused with HA tag were engineered onto EVs. Then, the EVs nanoparticle parameters were determined by NTA. The average size of the Con-EVs and E8-EVs was 103 ± 28 and 120 ± 16 nm, respectively (Fig. 1b), while the corresponding Zeta potentials (ζ) were − 24.3 ± 3.8 and − 24.1 ± 3.3 mV, respectively (Fig. 1c). Both of the engineered-EVs are physically similar. TEM indicated that the diameters for the most of particle EVs ranged from 30 to 120 nm, having negligible variations within morphology (Fig. 1d).

To confirm the specificity of E8-EVs against CDH17, we first knocked down the CDH17 protein in MKN45 cells with shRNA and then assessed the binding specificity of E8-EVs to MKN45 with/without CDH17 knockdown. As shown in Fig S2, knockdown CDH17 almost abolished the binding activity of E8-EVs to CDH17-positive cells; however, control shRNA which cannot disturb the expression of CDH17 did not influence the binding capability of E8-EVs to MKN45 cells. These data indicate that E8-EVs can specifically interact with CDH17 protein expressing on the surface of cancer cells. Next, to further prove that EVs could be efficiently internalized by CDH17 positive tumor cells, PKH67-labeled EVs were incubated with two gastric cancer cell lines MKN45 and IM95 at various time points, both of which were confirmed high expression of CDH17 protein [33]. Immunofluorescence staining revealed that EVs could be effectively internalized in MKN45 and IM95 cells in a time-dependent manner; nevertheless, fluorescence intensity of E8-EVs was superior to control EVs, which implied that internalization was enhanced due to the interaction between E8 nanobodies and membrane CDH17 protein. (Figure 1e and f). These results reveal that E8-EVs are capable of improving the targeting capacity towards CDH17-overexpressing cells, therefore promoting cellular uptake.

### Characterization of photophysical properties of CR@E8-EVs

**Fig. 2:**
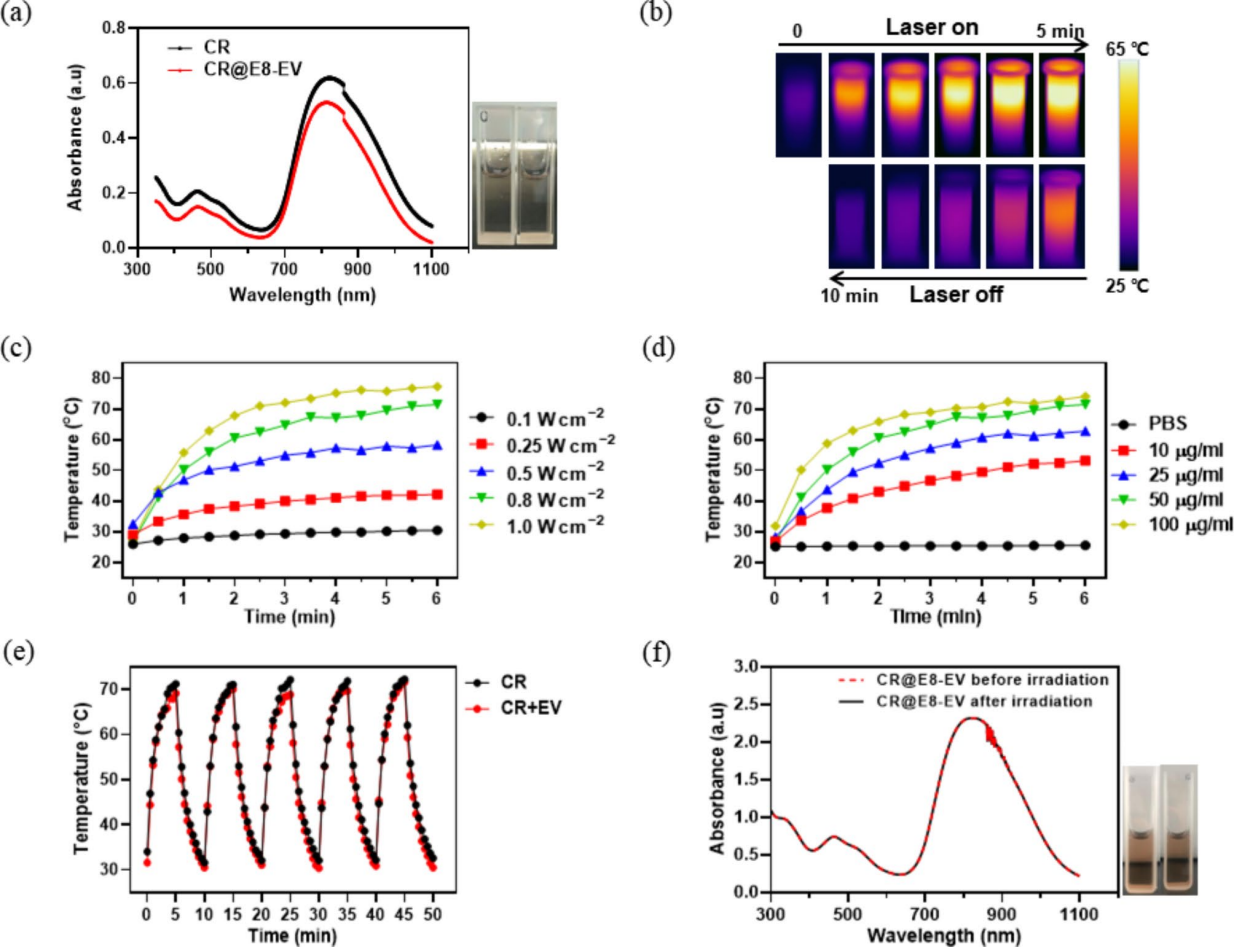
**(a)** UV − vis absorption spectra for CR (dispersed in DMSO, 12.5 µg/mL) and CR@E8-EVs (dispersed in water, 12.5 µg/mL). Insets are photographs of CR and CR@E8-EVs dispersion taken under bright light. **(b)** IR thermal images of the CR@E8-EVs (50 µg/mL based on CR) under 808 nm laser irradiation (0.8 W/cm^2^) for different durations. **(c)** Temperature variation of CR@E8-EVs with different laser power density (50 µg/mL dose). **(d)** Temperature variation of CR@E8-EVs with different concentrations treated with 808 nm, 0.8 W/cm^2^. **(e)** Photothermal curves for CR and CR@E8-EVs subjected to 808 nm laser irradiation on/off cycles at 0.8 W/cm^2^. **(f)** Absorption spectra and photos (insets) of CR@E8-EVs before and after 808 nm laser irradiation cycles (0.8 W/cm^2^)

Next, we encapsulated CR-DPA-T into the E8-EVs as described in the part 2.3, which was termed as CR@E8-EVs. To prove that EVs do not change the intrinsic characteristics of CR dye, we investigated the photophysical properties of CR@E8-EVs in solution state. As examined within UV–vis–NIR absorption spectrum (Fig. 2a), free CR and CR@E8-EVs displayed similar absorption pattern (400 ~ 1100 nm) centered at approximately 810 nm, matching well with the 808 nm laser. There had no difference of the absorption and color between the free CR and CR@E8-EVs (inset images in Fig. 2a). The amount and loading efficiency of CR encapsulated in the prepared CR@E8-EVs was determined by the calibration absorption curve of CR in water at 830 nm. The maximum absorption of CR increased linearly with the increasing concentration of CR (Fig. S1a, b), and the correlation coefficient was 1.0 (Fig. S1b). The concentration of CR in the CR@E8-EVs solution was calculated to be 4.6 µg/ml, thus the loading efficiency of CR in CR@E8-EVs was about 92% (4.6 * 100 / 500 * 100% = 92%) (Fig. S1). ]

Photothermal property and photothermal stability are two important parameters to evaluate the ability of PTAs. Subsequently, we explored in vitro photothermal function of CR@E8-EVs with an infrared camera. As depicted in the infrared images (Fig. 2b), upon 808 nm laser irradiation (0.8 W cm^2^), aqueous CR@E8-EVs temperature radically raised to approximately 70 °C within 5 min (ΔT ≈ 45 °C), exhibiting an obvious photothermal conversion process. Furthermore, it was showed that the temperature elevation of CR@E8-EVs solution was apparently correlated with the laser power density and the solution concentrations (Fig. 2c, d, S4). In addition to excellent photothermal property, CR@E8-EVs also exhibited desirable photothermal stability. Specifically, free CR and CR@E8-EVs (50 µg/mL) were exposed to 808 nm laser (0.8 W cm^− 2^) for five irradiation/cooling cycles, each of which contains irradiation (laser on) for 5 min and then cooling down (laser off) for 5 min. As indicated in Fig. 2e, upon cycling irradiation − cooling, the temperature elevation remained almost the same during the five irradiation − cooling cycles, demonstrating that EVs do not alter the stability of CR-DPA-T and CR-DPA-T possesses excellent thermal- and photo-stability. Moreover, the absorption spectrum of CR@E8-EVs showed negligible change after the five cycles, which further confirmed its outstanding photothermal stability (Fig. 2f). Taken together, these results indicate that encapsulated CR in engineered-EVs display the similar properties with free CR, implying that CR@E8-EVs is a promising candidate for targeted delivery of CR and in vivo PTT applications based on excellent photothermal performance of CR.

### In vitro photothermal activity of CR@E8-EVs

**Fig. 3:**
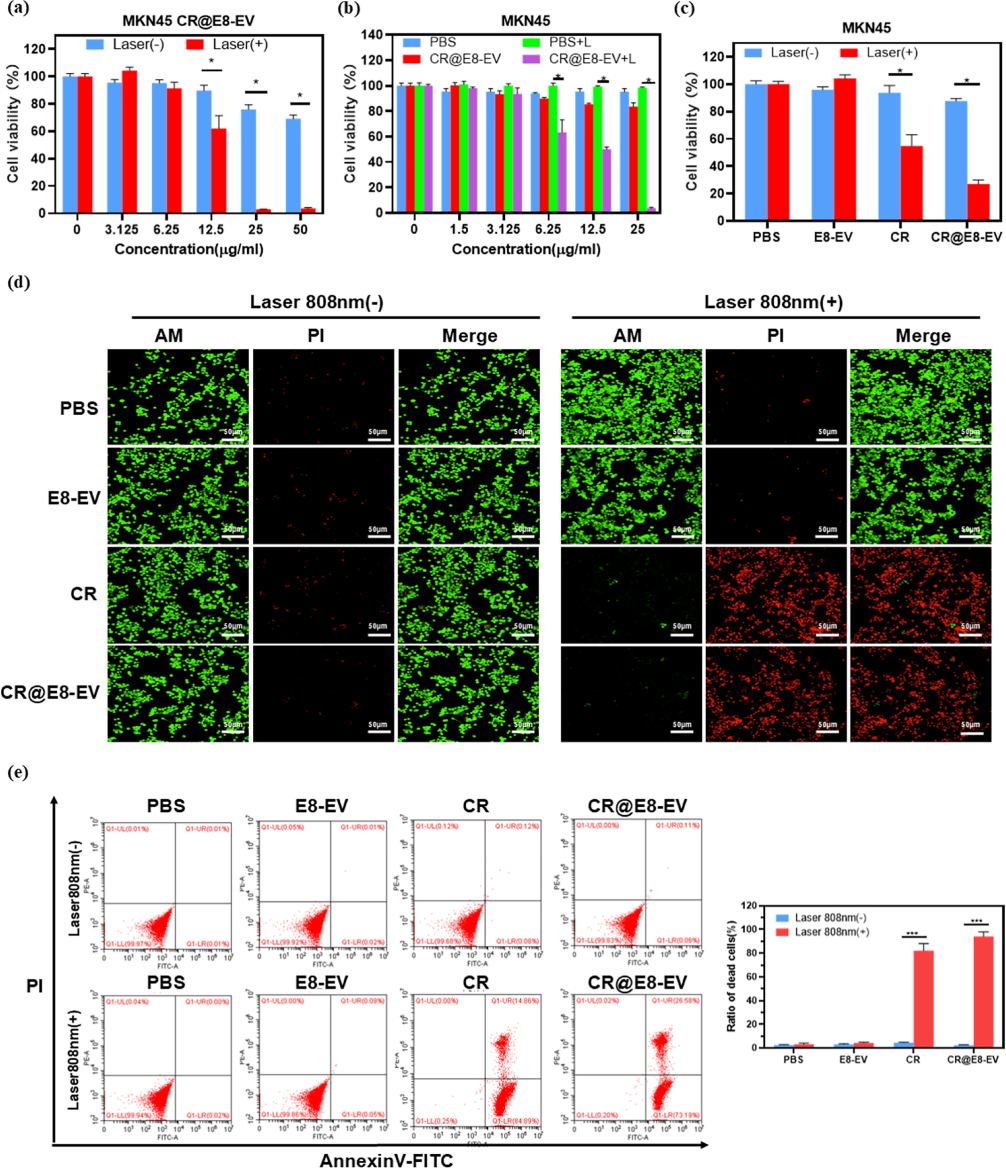
**(a)** The in vitro PTT cytotoxicity of CR@E8-EVs in MKN 45 cultures with/without 0.8 W/cm^2^ 808 nm laser irradiation for 10 min. Cell viability was identified through CCK8 assessment. **(b)** Cell viability of CR and PBS against MKN 45 cells and **(c)** relative viabilities for MKN 45 cells post-PBS (100 µL), E8-EVs (100 µL), CR (25 µg/mL, 100 µL) and CR@E8-EVs (25 µg/mL based on CR, 100 µL) therapy in the presence or absence of laser. **(d)** Fluorescence imaging for live/dead MKN45 cells (green/red) with Calcein-AM and PI staining following multiple therapies. NIR light irradiation (808 nm, 0.8 W cm^− 2^, 5 min) was performed once cells were placed into incubation with various treatment for 12 h (equal to 25 µg/mL CR). Scale bars, 50 μm. **(e)** Apoptosis and necrosis assessments through flow cytometry in MKN 45 cells after different treatments. Laser irradiation (808 nm, 0.8 W cm^− 2^, 5 min) was performed after cells were incubated with various drugs for 12 h (PBS, 100 µL; E8-EVs, 100 µL; CR, 25 µg/mL, 100 µL and CR@E8-EVs, 25 µg/mL based on CR, 100 µL)

Inspired by the great photothermal performance of CR@E8-EVs in vitro, we then evaluated the cytotoxicity and the ability of photothermal ablation of CR@E8-EVs on MKN45 cells. First, the inherent photo-toxicity of CR@E8-EVs was determined through standard CCK8 (cell counting kit-8) viability assessment. MKN45 cells were incubated with CR@E8-EVs under various concentrations (24 h). As depicted within Fig. 3a, no cytotoxicity of CR@E8-EVs to cells was observed even with the incubation concentration up to 50 µg/mL without irradiation, illustrating the excellent biocompatibility. On the contrary, under 808 nm laser irradiation (0.8 W cm ^− 2,^ 5 min), CR@E8-EVs showed remarkably photothermal cytotoxicity to cell viability which was dramatically reduced as concentration reached 25 µg/mL, at which cells were almost completely eliminated (Fig. 3a). Therefore, CR@E8-EVs exhibited high-efficient photothermal effect in cells. But for the control treatments as shown in Fig. 3b, no obvious toxic effects for PBS, PBS plus laser and CR@E8-EVs were observed under irradiation free condition, while remarkable reduction of cell viability was discovered for CR@E8-EVs plus laser. On the other hand, as shown in Fig. 3c, cells were negligibly affected in the four groups without irradiation. However, the cell viabilities in the groups of free CR and CR@E8-EVs with laser-irradiation were significantly decreased. Moreover, cell viability that received treatment with CR@E8-EVs was lower than that of free CR, indicating that the CR dye functionalized with engineered EVs was increased in the uptake of cultures due to high tumor targeting ability compared with free CR.

To further visualize the photothermal ablation capability of CR@E8-EVs, we subsequently conducted Calcein-AM and PI (propidium iodide) staining of MKN45 cells through confocal scanning microscopy (Fig. 3d). For the unirradiated groups of PBS, E8-EVs, CR and CR@E8-EVs, the majority of cells showed widespread green fluorescence, implying no dark cytotoxicity of CRs. In contrast, when irradiating the cells with a 808 nm laser for 5 min, the free CR and CR@E8-EVs induced obvious cell death as shown with the red fluorescence, validating the high PTT efficiency for CRs.

Additionally, the flow cytometry assessment through Annexin V-FITC (fluoresceine isothiocyanate)/PI (propidium iodide) double staining was used to detect pro-apoptotic effect for CR dye with or without irradiation. The results in Fig. 3e indicated that negligible apoptosis and necrosis were induced in all groups without irradiation, suggesting the low toxicity of the CR dye. While the groups of CR plus laser and CR@E8-EVs plus laser showed apparent cell apoptosis of MKN45 cells. In comparison, the apoptotic rate of CR@E8-EVs plus laser was slightly higher than the free CR plus laser (Fig. 3e). These results were consistent with that of CCK8, further confirming the PTT capacity of CR@E8-EVs.

### In vivo targeting ability of E8-EVs

**Fig. 4:**
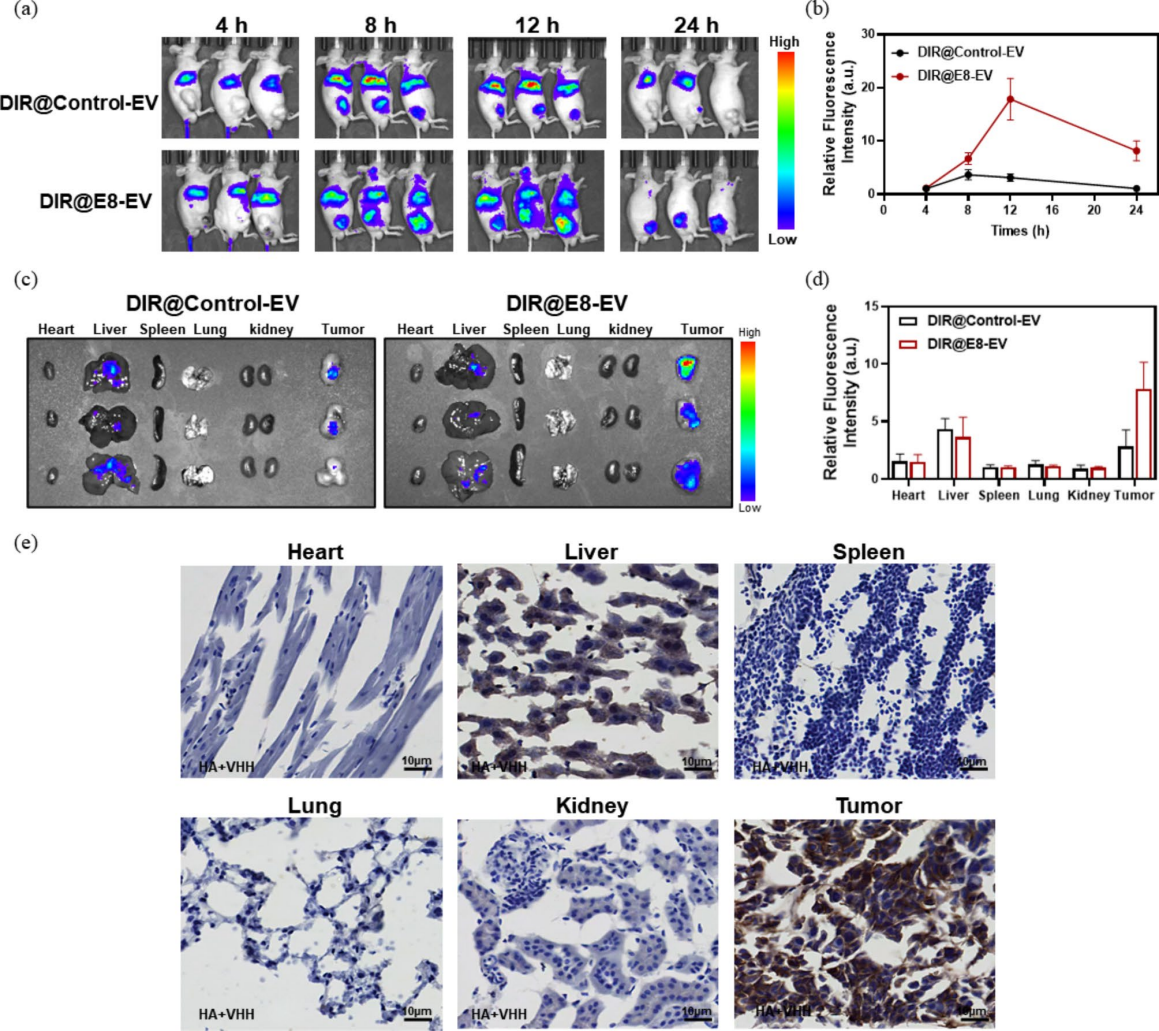
**(a)** Whole body optical imaging and **(b)** quantification analysis of MKN45 tumor bearing mice at different postinjection timepoints. **(c)** Ex vivo optical imaging and **(d)** quantification analysis of major organs dissected from in vivo imaged mice. **(e)** E8-EVs tissue distribution after 24 h injection. Scale bars, 10 μm, n = 3

After proved the in vitro photothermal activity of CR@E8-EVs, we next investigated the in vivo targeting ability of E8-EVs by using the subcutaneous MKN45 xenograft tumor model. To visualize the in vivo performance of E8-EVs, a commercially available contrast agent, DIR, was harnessed to label E8-EVs (termed DIR@E8-EVs). DIR is a useful agent in live imaging or biomaterial tracing because the emitted infrared light from DIR can efficiently pass through cells and tissues with low background fluorescence in the infrared light range. The biodistribution and accumulation were constantly tracked by an in vivo imaging system (IVIS) at pre-set timepoints after injection of DIR@E8-EVs and DIR@Con-EVs to the mice bearing MKN45 tumors (corresponding DIR dose: 1 mg/kg). As depicted in Fig. 4a, tumor fluorescence was clearly detected during 4 to 8 h post-injection for both groups of DIR@E8-EVs and DIR@Con-EVs. But the DIR@E8-EVs exhibited much higher signals than that of DIR@Con-EVs due to the active tumor-targeting ability, and the intensity was increased with time, reaching peak at 12 h for both groups. Thereafter, the imaging signals at tumor sites were decreased from 12 to 24 h post-administration. Nevertheless, fluorescence signals within tumors treated with DIR@E8-EVs were still clear and strong even after 24 h as compared with DIR@Con-EVs, which were undetectable in the tumors after 24 h, and only seen weak signals in the livers (Fig. 4b). Ex vivo imaging of dissected tumors and main organs was further conducted to confirm the EVs distribution. Signal intensity of DIR@E8-EVs within tumors was much higher in comparison to DIR@Con-EVs. The signal in the control organs was not observed except livers (Fig. 4c). To verify the specificity of E8-EVs against CDH17-positive tumors, another CDH17-negative 4T1 tumor model was further used to test the imaging ability of E8-EVs (Figure S5). The results indicated that E8-EVs exhibited similar tumor imaging ability to control-EVs irrespective of in vivo imaging or ex vivo imaging in 4T1 tumor-bearing mice, implying that E8-EVs could only exert the efficient payload delivery in CDH17-overexpressing tumors. Further immunohistological analysis to vital control organs and tumors in the DIR@E8-EVs group implied that no nanobody positive staining was observed in the most of control organs except liver tissues, which displayed weak nanobody staining due to non-specific phagocytosis of reticuloendothelial system; strong nanobody staining was identified in the tumors, indicating that DIR@E8-EVs could effectively targeted CDH17-positive tumors (Fig. 4e). All these results reveal that the E8-EVs is capable of delivering CR dye for in vivo tumor targeting PTT.

### In vivo photoacoustic imaging ability of CR@E8-EVs

**Fig. 5:**
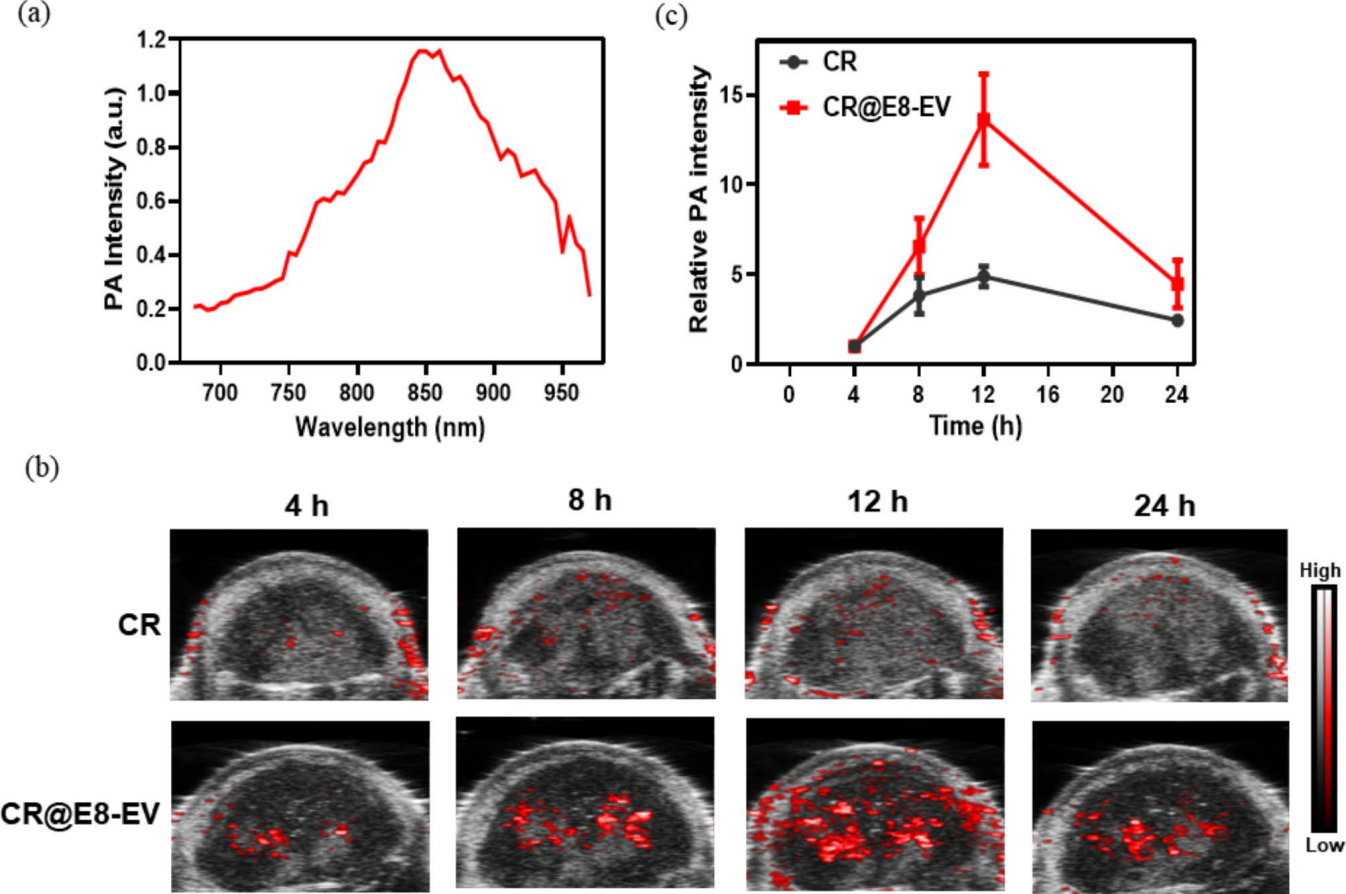
**(a)** PA spectrum of CR@E8-EVs (50 µg/mL based on CR) in water. **(b)** In vivo PA imaging and **(c)** PA signal intensity of MKN45 tumor at 4, 8, 12, 24 h post-tail vein injection of CR (50 µg/mL) and CR@E8-EVs (50 µg/mL based on CR)

PA imaging property as the symbiotic function for photothermal agents, can reveal the profile of tumor tissues with high spatial resolution [38]. Croconaine dyes have been reported for efficient PAI applications with ultrahigh chemical-, thermal- and photo-stability [19, 39–42]. Therefore, we further evaluated the PAI property of the CR@E8-EVs on the MKN45 tumor-bearing mice. The PA images and intensity were monitored at varied post-injection times of CR@E8-EVs; simultaneously, the free CR was also conducted as a control. The PA spectrum of CR@E8-EVs exhibited a broad band (700–950 nm) covering NIR-I region with a maximum at about 850 nm (Fig. 5a). Hence, the in vivo PA imaging was conducted at 850 nm. As depicted in Fig. 5b, PA signals in both free CR and CR@E8-EVs became clearly detectable at 4 h after injection, presumably thanks to enhanced permeability and retention (EPR) effect. Signals for free CR from the tumor sites were increased slightly over time and backed to the background level at 24 h, while the PA signals of CR@E8-EVs presented significant increase due to the active tumor-targeting ability; the signals peaked at 12 h post-injection and then decreased, implicating that 12 h post-injection was the optimal timepoint for PAI and tumor PTT treatment (Fig. 5b, c).

### Imaging-driven photothermal treatment in vivo

**Fig. 6:**
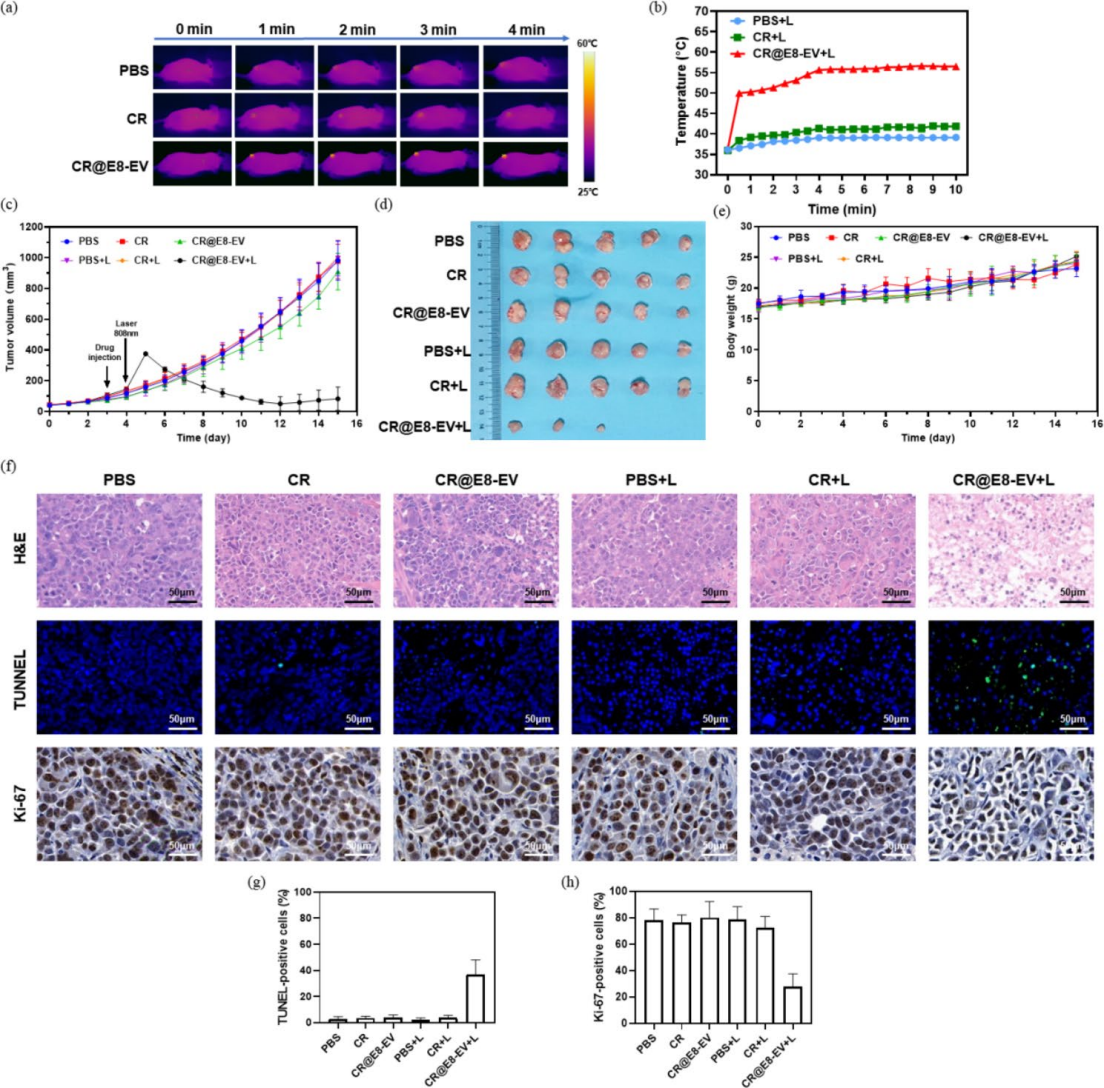
**(a)** Photothermal imaging and **(b)** tumor temperature changes under 808 nm laser irradiation. **(c)** Tumor growth curves following various therapies. **(d)** Tumor dissection photographs for all six groups (n = 5) at day 16 post-therapy. **(e)** Body weight of mice during treatment. **(f)** Histological H&E, and fluorescence TUNEL and Ki67 staining for tumor tissues at the end of the treatment (scale bars, 50 μm). **(g)** Quantification of TUNEL-positive cells. **(h)** Quantification of Ki67 positive cells

Encouraged by the excellent in vivo photothermal effect, superb tumor targeting performance and PA imaging ability of CR@E8-EVs, we next performed the photothermal treatment in vivo with the MKN45 tumor xenograft model and an 808 nm laser irradiation. Tumor-bearing nude mice were stochastically separated within six cohorts (n = 5): (1) PBS, (2) PBS plus laser, (3) CR, (4) free CR plus laser. (5) CR@E8-EVs and (6) CR@E8-EVs plus laser. For PBS, CR and CR@E8-EVs groups, 100 µL PBS, free CR and CR@E8-EVs (50 µg/mL based on CR) were injected within tumor-bearing mice through tail vein without laser irradiation after tumor volumes approached approximately 100 mm^3^. For PBS plus laser, CR plus laser and CR@E8-EVs plus laser groups, the tumors in these groups were continuously irradiated with 808 nm laser (0.8 m W/cm^2^) for 10 min after identical dose injection. Here, the irradiation was administered 12 h after injection according to the results of NIR/PA imaging, at which time point the maximal tumor accumulation was observed. IR thermal imaging of three irradiation groups (PBS, free CR and CR@E8-EVs) was recorded through monitoring real-time temperature changes in tumors under laser irradiation (Fig. 6a,b). As illustrated in IR thermal images, tumor temperature for control groups treated with only PBS and free CR demonstrated a slight increase from 37 to 41 °C after the laser irradiation, implying that continuous 808 nm laser irradiation with 0.8 W cm^− 2^ has negligible heat-causing effect and that free CR could not effectively accumulate into tumor tissues, which is incapable of inducing obvious temperature elevation. On the contrary, the tumor temperature for the CR@E8-EVs plus laser group at the irradiated area rapidly increased to over 50 °C within 40s, which was sufficient to inhibit tumor growth effectively. These data further indicate that CR@E8-EVs is an effective PTA with good penetration and high photothermal conversion.

To quantitatively assess the photothermal ablation efficacy of each group, tumor dimensions and mouse body weight were continuously monitored daily post-therapy. It was found that the tumors grew rapidly without any inhibitory effects in the control groups (PBS and PBS plus laser) during the treatment period, which indicated that 808 nm laser irradiation alone cannot inhibit tumor growth. Meanwhile, the tumor growth in the free CR and CR plus laser groups was similar to PBS (Fig. 6c), illustrating that CR itself possesses no targeting ability to cancer in vivo and cannot result in temperature elevation after irradiation. The tumor growth in the mice received the treatment with CR@E8-EVs plus laser was significantly suppressed during the whole process, indicating that E8-EVs could effectively deliver the CR dye to tumor tissues so that accumulated CR dye in tumor sites could initiate powerful PTT potency to control tumor progression (Fig. 6c). By the end of the treatment procedure, all the mice were sacrificed and tumors were photographed (Fig. 6d). The tumor images showed the same trend with the volume, and the average tumor size in the CR@E8-EVs plus laser group was significantly reduced with a relatively small tumor volume and even two tumors disappeared (Fig. 6d). Photothermal therapy as a therapeutic modality can not only directly ablate tumors but also provoke the anti-tumor immunity and reprogram tumor microenvironment through the induction of immunogenic cell death (ICD), even induce long-term immune memory, which has been validated in competent mouse tumor models [43–47], which might the main cause in current study for tumor eradication in two mice where macrophages or dendritic cells could be intensely activated in our compromise tumor model and phagocytosis might be enhanced. Further investigation is need to decipher the changes in anti-tumor immunity and tumor microenvironment in competent mouse tumor model after CR-mediated PTT. These tumor inhibition findings were consistent with the results of in vitro experiments, which further verifies the excellent PTT capacity of CR@E8-EVs. Additionally, no apparent body-weight loss and clinical abnormality were observed in all mice in these six groups over the 15-day treatment period, suggesting that photothermal treatment has good in vivo biosafety (Fig. 6e).

To further evaluate the effect of tumor inhibition and the biosafety of PTA, histopathological analysis of tumors and major control organs (heart, liver, spleen, lungs, kidneys and colons) was performed through standard hematoxylin and eosin (H&E) staining after therapy. No noticeable tissue damage/abnormality and inflammatory lesion of the control organs were identified from all the groups as shown in Fig. S6, demonstrating the favorable biosafety of CRs/EVs-based PTT. Furthermore, H&E staining analysis for tumor tissues revealed that CR@E8-EVs plus laser irradiation showed severe cell damage with more sparse tissues, whereas no obvious damage was observed in the other groups (Fig. 6f). Moreover, TUNEL analysis for cell apoptosis assessment unraveled that CR@E8-EVs plus laser treatment induced significant cell apoptosis compared with the other groups; and further Ki67 staining indicated that tumor cell proliferation was substantially inhibited in CR@E8-EVs plus laser group compared with control groups (Fig. 6f). All of the results above implicate that CR@E8-EV- based PTT could exert superb anti-tumor potency and engineered EVs is an effective strategy for the delivery of CR dyes to produce prominent photothermal effect on tumors.

## Conclusions

In the current study, we designed and fabricated an efficient bio-nanocomposite (CR@E8-EVs) with active tumor targeting capability through the integration of CR-DPA-T and CDH17 nanobody-engineered EVs. CR@E8-EVs simultaneously consolidated the merits of CRs and EVs, which showed outstanding biocompatibility, high photostability, excellent tumor targeting capability, high PCE and good biosafety. The in vitro studies demonstrated high efficient photothermal cytotoxicity to CDH17-positive cancer cells. In vivo imaging investigation manifested that CR@E8-EVs could act as a biocompatible and effective PAI agent for tumor diagnosis. Meanwhile, single-dose injection and one time irradiation could markedly ablate tumors owing to the excellent targeting ability and intrinsically superior photothermal performance of CR@E8-EVs; PTT possesses the capability of reprogramming tumor microenvironment and induced long-term anti-tumor immunity [46, 47], and further investigation is need to explore the changes in anti-tumor immunity and tumor microenvironment and design new combinational strategies with immune checkpoint blockades in competent mouse tumor model after CR-mediated PTT. Collectively, this platform consisting of CDH17 nanobody-engineered EVs and CR dyes provides a novel tumor theronostic system, and holds great promise for clinical translation. Such work offers an effective method to fabricate novel phototheranostic systems, opening new avenues for cancer theranostics.

## Electronic supplementary material

Below is the link to the electronic supplementary material.

Supplementary Material 1

## Data Availability

The data and materials of the study are included in this article and its supplementary information files.
