# Correction: Targeted delivery of organic small-molecule photothermal materials with engineered extracellular vesicles for imaging-guided tumor photothermal therapy

**DOI:** 10.1186/s12951-026-04073-2

**Published:** 2026-03-02

**Authors:** Yafang Dong, Peng Xia, Xiaolong Xu, Jing Shen, Youbin Ding, Yuke Jiang, Huifang Wang, Xin Xie, Xiaodong Zhang, Weihua Li, Zhijie Li, Jigang Wang, Shan-Chao Zhao

**Affiliations:** 1 Department of Urology, the Third Affiliated Hospital of Southern Medical University, Guangzhou, 510500 Guangdong P. R. China; 2 Department of Nephrology, Shenzhen Key Laboratory of Kidney Diseases, Shenzhen Clinical Research Centre for Geriatrics, Shenzhen People’s Hospital, The First Affiliated Hospital, Southern University of Science and Technology, Shenzhen, 518020 Guangdong P. R. China; 3 Department of Hepatobiliary & Pancreatic Surgery, Zhongnan Hospital of Wuhan University, Wuhan, 430072 Hubei P. R. China; 4 Department of Oncology, Department of Infectious Disease, Shenzhen People’s Hospital, The First Affiliated Hospital, Southern University of Science and Technology, Shenzhen, 518020 Guangdong P. R. China; 5 Department of Medical Imaging, The Third Affiliated Hospital of Southern Medical University, Guangzhou, 510630 Guangdong P. R. China; 6 Medical imaging department, Shenzhen Second People’s Hospital/ the First Affiliated Hospital, Shenzhen University Health Science Center, Shenzhen, 518035 Guangdong P. R. China; 7 State Key Laboratory for Quality Ensurance and Sustainable Use of Dao-di Herbs, Artemisinin Research Center, Institute of Chinese Materia Medica, China Academy of Chinese Medical Sciences, Beijing, 100700 P. R. China; 8 Department of Oncology, the Affiliated Hospital of Southwest Medical University, Luzhou, Sichuan P. R. China; 9 Department of Urology, Nanfang Hospital, Southern Medical University, Guangzhou, 510515 Guangdong P. R. China

**Correction: Journal of Nanobiotechnology (2023) 21:442**

10.1186/s12951-023-02133-5

After publication, the authors identified an inadvertent error in Fig. 3. Specifically, in Fig. 3d, the merged and Calcein-AM–treated images (AM and Merge panels) of the PBS and E8-EV groups under laser irradiation (“Laser808 nm (+)” panel) were unintentionally duplicated during figure assembly. Importantly, this error does not affect the interpretation, data validity, or conclusions of the study, as both the PBS and E8-EV groups showed no detectable cytotoxicity under these conditions.

For completeness and transparency, the correct and old incorrect versions are displayed below.

Incorrect Fig. 3:


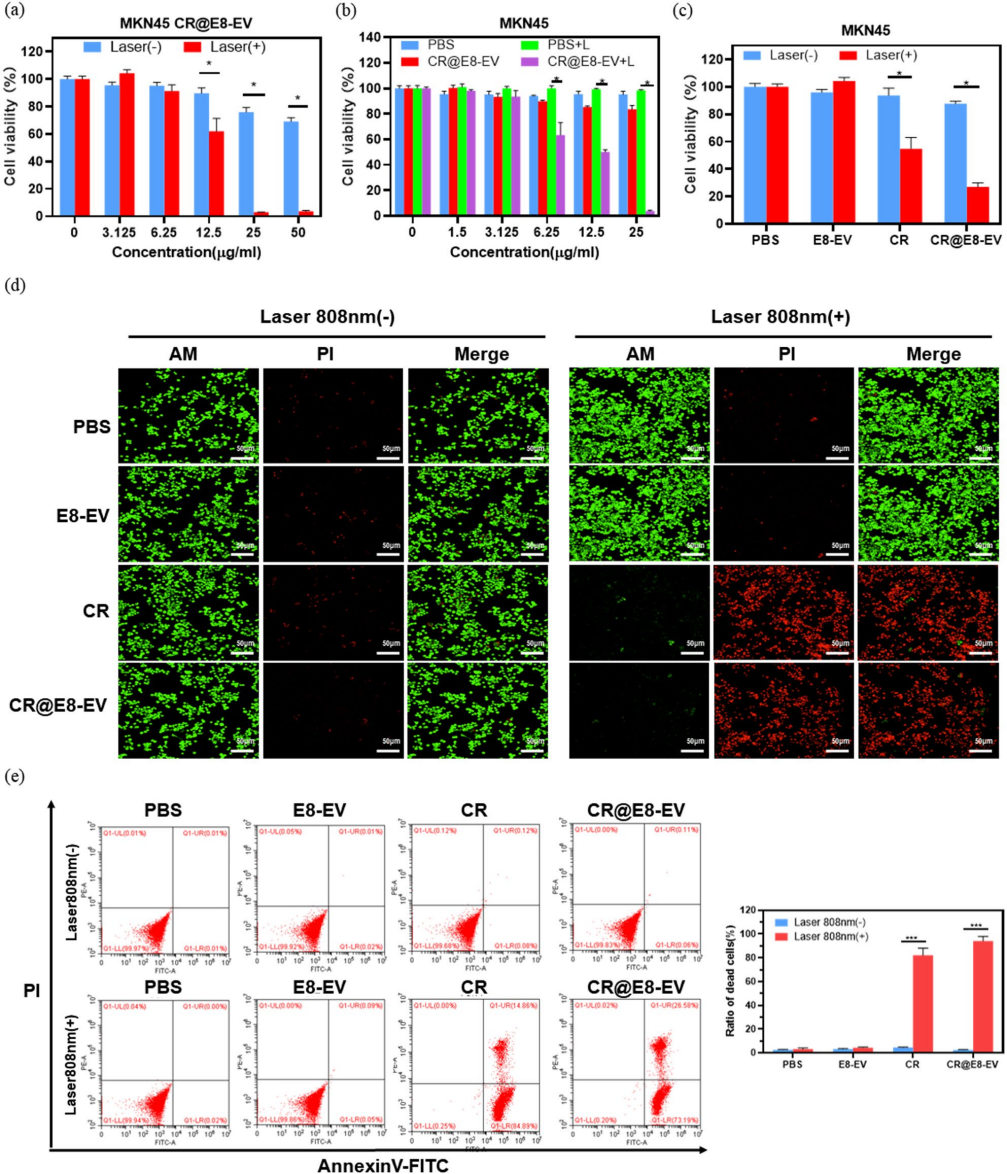


Correct Fig. 3:

**Fig. 3:**
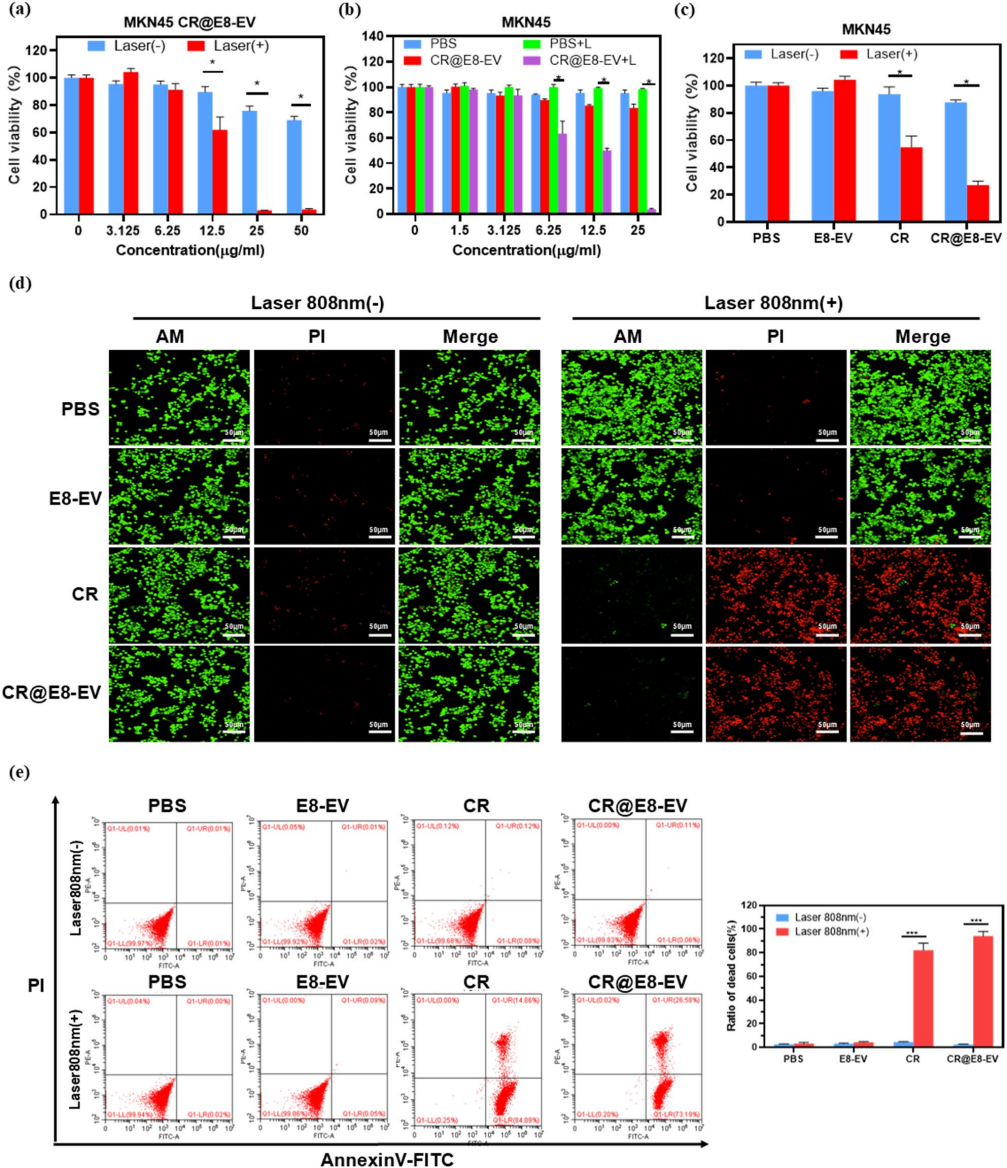
(**a**) The in vitro PTT cytotoxicity of CR@E8-EVs in MKN 45 cultures with/without 0.8 W/cm^2^ 808 nm laser irradiation for 10 min. Cell viability was identified through CCK8 assessment. (**b**) Cell viability of CR and PBS against MKN 45 cells and (**c**) relative viabilities for MKN 45 cells post-PBS (100 µL), E8-EVs (100 µL), CR (25 µg/mL, 100 µL) and CR@E8-EVs (25 µg/mL based on CR, 100 µL) therapy in the presence or absence of laser. (**d**) Fluorescence imaging for live/dead MKN45 cells (green/red) with Calcein-AM and PI staining following multiple therapies. NIR light irradiation (808 nm, 0.8 W cm^− 2^, 5 min) was performed once cells were placed into incubation with various treatment for 12 h (equal to 25 µg/mL CR). Scale bars, 50 μm. (**e**) Apoptosis and necrosis assessments through flow cytometry in MKN 45 cells after different treatments. Laser irradiation (808 nm, 0.8 W cm^− 2^, 5 min) was performed after cells were incubated with various drugs for 12 h (PBS, 100 µL; E8-EVs, 100 µL; CR, 25 µg/mL, 100 µL and CR@E8-EVs, 25 µg/mL based on CR, 100 µL)

The original article has been corrected.

