# Supplementary material for: Targeted delivery of organic small-molecule photothermal materials with engineered extracellular vesicles for imaging-guided tumor photothermal therapy
Source: J Nanobiotechnology. 2023 Nov 23;21:442. doi: 10.1186/s12951-023-02133-5 (PMC10666357; doi:10.1186/s12951-023-02133-5)
Supplement: Supplementary file 1 — Supplementary Material 1 [file 12951_2023_2133_MOESM1_ESM.docx]

**Targeted Delivery of Organic Small-Molecule Photothermal Materials with Engineered Extracellular Vesicles for Imaging-Guided Tumor Photothermal Therapy**

Yafang Dong^1, 2, #^, Peng Xia^2, 3, #^, Xiaolong Xu^2, #^, Jing Shen^4, #^, Youbin Ding^5^, Yuke Jiang^2^, Huifang Wang^2^, Xin Xie^2^, Xiaodong Zhang^5^, Weihua Li^6,*^, Zhijie Li^2, *^, Jigang Wang^1,2, 7,8*^, Shan-chao Zhao^1, 9, *^

^1^Department of Urology, the Third Affiliated Hospital of Southern Medical University, Guangzhou, 510500, Guangdong, P. R. China.

^2^Department of Nephrology, Shenzhen Key Laboratory of Kidney Diseases, and Shenzhen Clinical Research Centre for Geriatrics, Shenzhen People’s Hospital, The First Affiliated Hospital, Southern University of Science and Technology, Shenzhen 518020, Guangdong, P. R. China.

^3^Department of Hepatobiliary & Pancreatic Surgery, Zhongnan Hospital of Wuhan University, Wuhan, 430072, Hubei, P. R. China

^4^Department of Oncology, and Department of Infectious Disease, Shenzhen People's Hospital, The First Affiliated Hospital, Southern University of Science and Technology, Shenzhen, 518020, Guangdong, P. R. China.

^5^Department of Medical Imaging, The Third Affiliated Hospital of Southern Medical University, Guangzhou, 510630, Guangdong, P. R. China.

^6^Medical imaging department, Shenzhen Second People’s Hospital/the First Affiliated Hospital of Shenzhen University Health Science Center, Shenzhen, 518035, Guangdong, P. R. China.

^7^State Key Laboratory for Quality Ensurance and Sustainable Use of Dao-di Herbs, Artemisinin Research Center, and Institute of Chinese Materia Medica, China Academy of Chinese Medical Sciences, Beijing 100700, P. R. China

^8^Department of Oncology, the Affiliated Hospital of Southwest Medical University

^9^Department of Urology, Nanfang Hospital, Southern Medical University, Guangzhou, 510515, Guangdong, P. R. China.

^#^These authors equally contribute to this work and share the first authorship.

*Corresponding authors:

[jgwang@icmm.ac.cn](mailto:jgwang@icmm.ac.cn)(Jigang Wang);

18804511716@163.com(Weihua Li)

[li.zhijie@szhospital.com](mailto:li.zhijie@szhospital.com)(Zhijie Li);

[lulululu@smu.edu.cn](mailto:lulululu@smu.edu.cn)(Shanchao Zhao)

**General information**

Croconic acid, n-butanol, toluene, and DMSO were commercially available and used as received without further purification unless otherwise specified. N,N-bis(4-methoxyphenyl)thiophen-2-amine (**S1**) was synthesized according to the reported protocol with quantitative yield.^[1]^ MKN 45, IM95 and 4T1 cells were obtained from Cell Bank of the Chinese Academy of Sciences (Shanghai, China), HEK-293 were obtained from FuHeng BioLogy (Shanghai, China). DMEM medium, RPMI1640 medium, fetal bovine serum (FBS), Exosome-depleted Fetal Bovine Serum, penicillinstreptomycin (Pen-Strep), and Lipofectamine™ 3000 Transfection Reagent were obtained from Thermo Fisher Scientific (Waltham, MA, USA). Cell Counting Kit-8 (CCK-8) was purchased from Dojindo Laboratories (Kumamoto, Japan). Annexin V-FITC/PI apoptosis kit was obtained from MultiSciences (Hangzhou, China). Apoptosis detection assay was analyzed using a flow cytometer (CytoFLEX; Beckman Coulter, Inc.) and the data were analyzed using FlowJo software (FlowJo, LLC). Confocal laser scanning microscopy (CLSM) characterization was carried out on a confocal laser scanning microscope (TCS SP8, Leica, Germany). Paraformaldehyde (PFA) (4%) was purchased from Biyuntian Company (Shanghai, China). PKH67 Green Fluorescent Cell Linker Mini Kit, Tunnel staining kit, BCA protein assay kit, and G418 was obtained from Sigma-Aldrich (St. Louis, MO, USA). Cell membrane NIR fluorescent probe DIR was purchased from Xi’an Ruixi Biological TechnologyCo.,Ltd. EV isolation was performed on a Beckman ultracentrifuge Floor-standing Ultracentrifuge (Optima XPN-100) Ultracentrifuge tubes were purchased from Beckman (Fullerton, California, USA). NTA: Malvern Instruments, U.K. TEM: JEOL JEM-1400 Plus, JEOL, Tokyo, Japan. Slide-A-Lyzer Mini Dialysis devices were supplied from Repligen Corporation (Waltham, MA, USA). Utra-100K was purchased from Millipore (Billerica, MA, USA). All reactions were carried out under dry nitrogen by using Schlenk techniques. Reaction progress was monitored by thin-layer chromatography (TLC) on silica plates (250 µmol/L thickness, bought from Qingdao Haiyang Chemical Co.) and spots were visualized by UV254 and 365 fluorescent indicator. Flash column chromatography was carried out using silica gel (100 mesh) bought from Aladdin. The ^1^H NMR and ^13^C NMR spectra were collected on a Bruker ARX 400 and 600 MHz spectrometer. The mice were kept anesthetized using 2% isoflurane in oxygen during the in vivo experiments.

**Synthesis**^[2]^

**3,5-Bis(5-(bis(4-methoxyphenyl)amino)thiophen-2-yl)cyclopentane-1,2,4-trione (CR-DPA-T)**. **S1** (0.436 g, 1.4 mmol, 2.0 eq), croconic acid (0.100 g, 0.7 mmol, 1.0 eq) were added into a 50 mL two-necked round-bottom flask with magnetic stirrer and a reflux condenser. The flask was degassed and flushed with nitrogen three times. Sequentially, 15 mL mixture of toluene/1-butanol (v/v, 1/1) was injected into the flask, and the reaction mixture was refluxed at 120 ^o^C for 3 h under nitrogen atmosphere. Afterwards, the solvent was evaporated and the crude product was purified by silica-gel column chromatography by using DCM/methanol (200:1 to 50:1) as eluent to give CR-DPA-T (0.142 g) in 28% yield.

Construction of CDH17 knockdown cell lines

CDH17 knockdown cell lines were established with interference sequence (5’-CGGGAGAGACAGATGGTATAT-3’) targeting CDH17 and negative control sequence of shRNAThe sequences were inserted into the shRNA backbone pLKO.1 lentiviral vector. The constructed plasmids were co-transfected with packaging vectors pSPAX2 and pMD2G into 293T-17 cells at a ratio of 2:1:1, and the viral supernatants were collected to transduce MKN45 cells for 48 h. The positive cells were screened with 2 μg/ml puromycin, and cellular proteins were extracted to verify the knockdown efficiency by western-blot or CDH17 was detected by flow cytometry after three consecutive generations of screening.

*In vivo* imaging for 4T1 tumors

For in vivo NIR II imaging, IR783@control-EVs and IR783@E8-EVs (1 mg/kg) were intravenously administered within 4T1 tumor-bearing mice. Imaging was recorded and monitored with real-time fluorescent signal changes across specific timepoints (4, 8, 12, and 24 h) through NIR-II *in vivo* imaging system MARS (Artemis Intelligent Imaging, Shanghai).


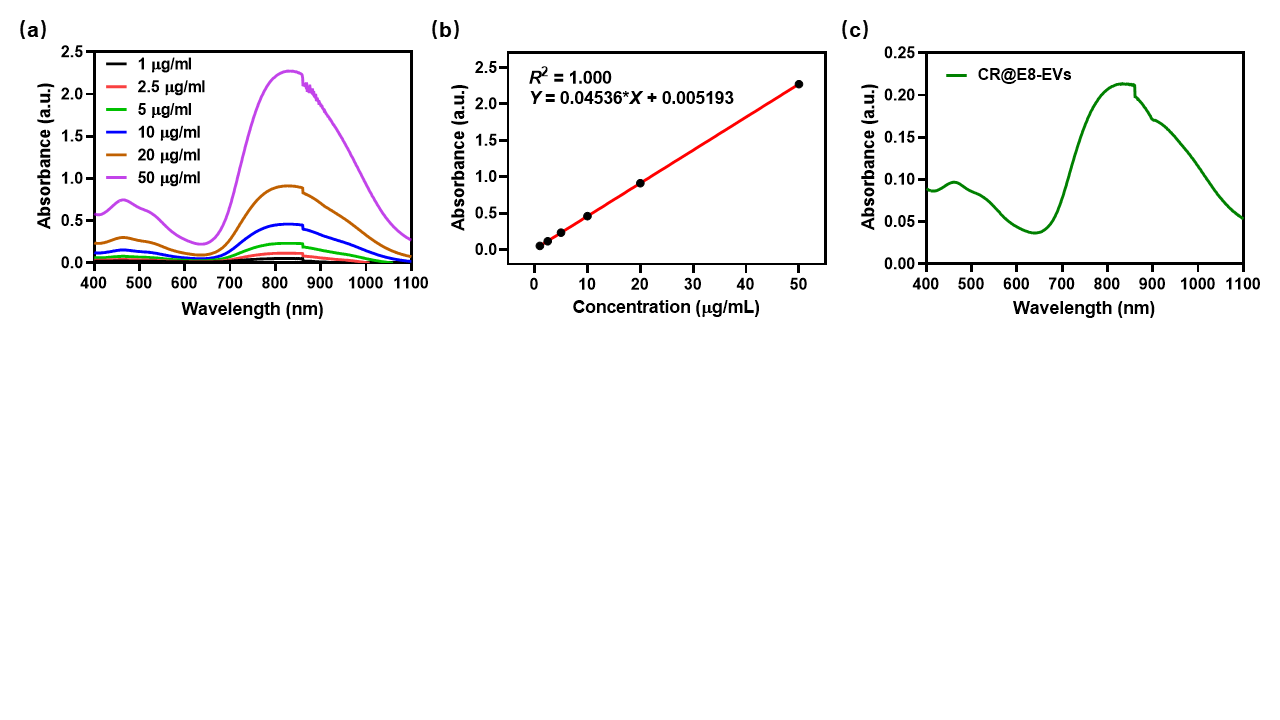


**Fig. S1.** (a) UV absorption spectra of CR in water at different concentrations (1, 2.5, 5, 10, 20, and 50 μg/mL). The water solution of CR was obtained by mixing 50 μL of CR DMSO solution (1 mg/mL) with 950 μL deionized water uniformly. (b) The fitted calibration curve of concentration vs absorption of CR solution at 830 nm. (c) The absorption spectrum of as-prepared CR@E8-EVs diluted 100 times. The concentration of CR in the CR@E8-EVs solution was calculated to be 4.6 μg/mL, thus the loading efficiency of CR in CR@E8-EVs was about 92% [4.6 * 100 / 500 * 100% = 92%].


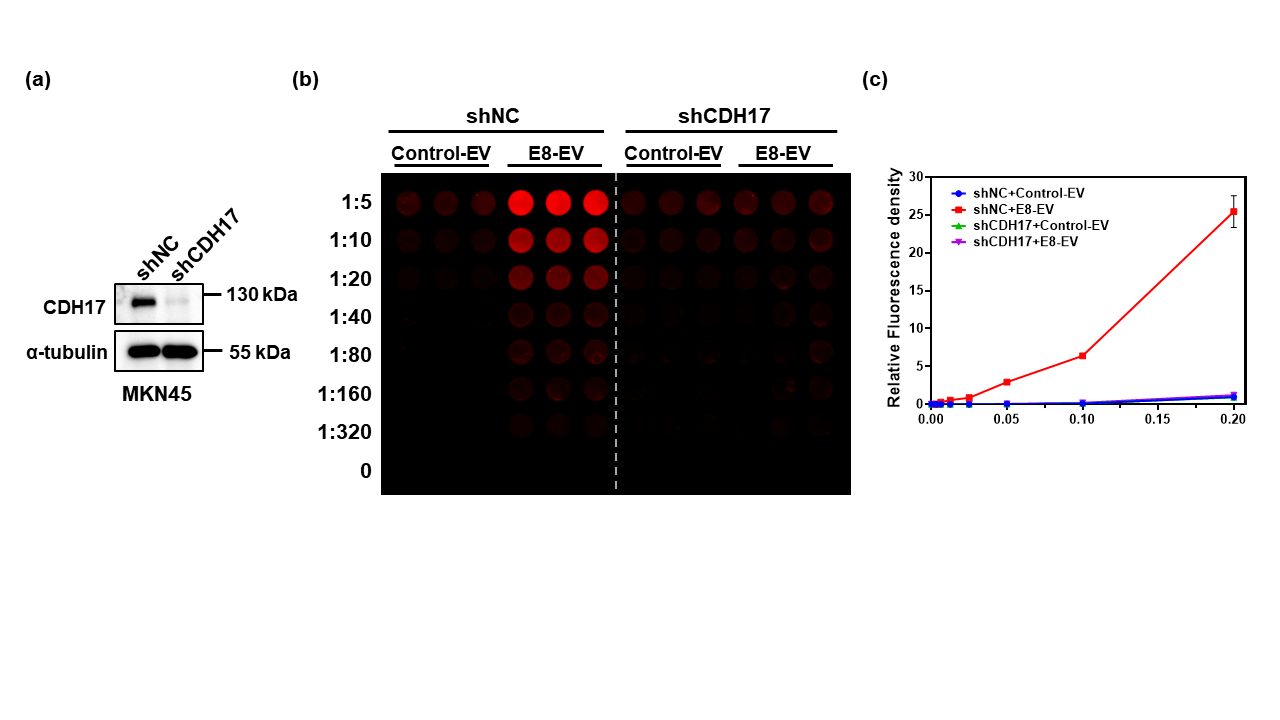


**Fig. S2.** (a) Validation of knockdown CDH17 with shRNA in MKN45 cells determined with western blot. (b) Binding specificity and (c) quantification of control and E8 nanobody to CDH17 in CDH17-knockdown cell lines assessed by fluorescent cell ELISA (n=3). Data are expressed as mean±SEM.


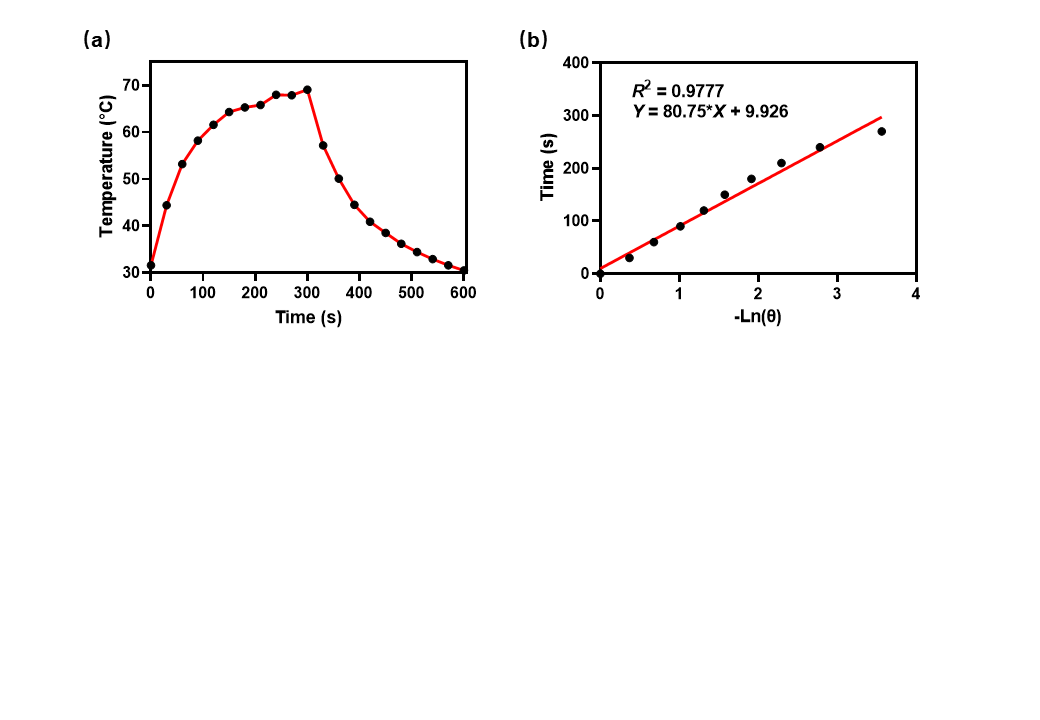


**Fig. S3.** (a) The heating curve of the CR@E8-EVs aqueous dispersion in a procedure of laser-on and off (808 nm, 0.8 W cm-2 laser was used). (b) The linear cooling time data versus -ln(θ) obtained from the cooling period of (a).


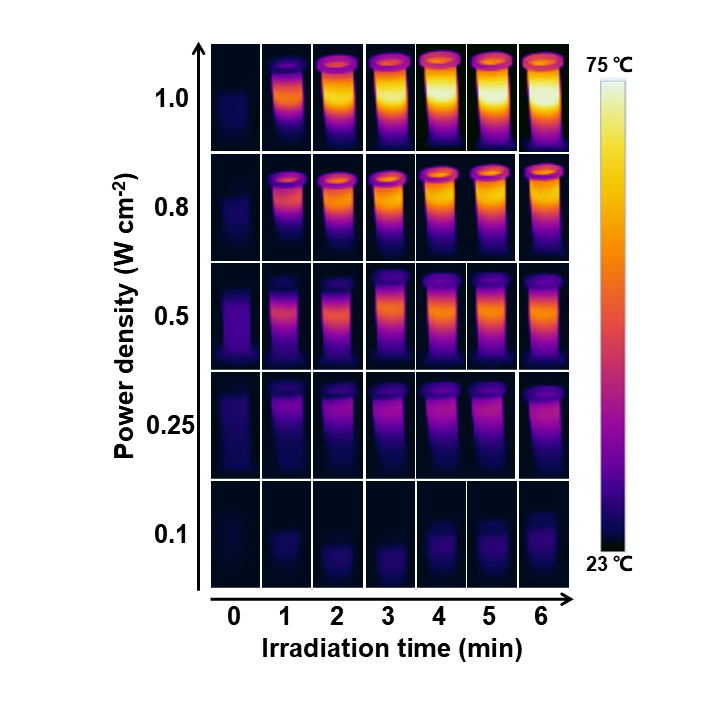


**Figure S4.** Infrared thermal images of CR@E8-EVs (50 μg/mL based on CR) upon exposure to the NIR laser (808 nm, 6 min) at different power intensity.


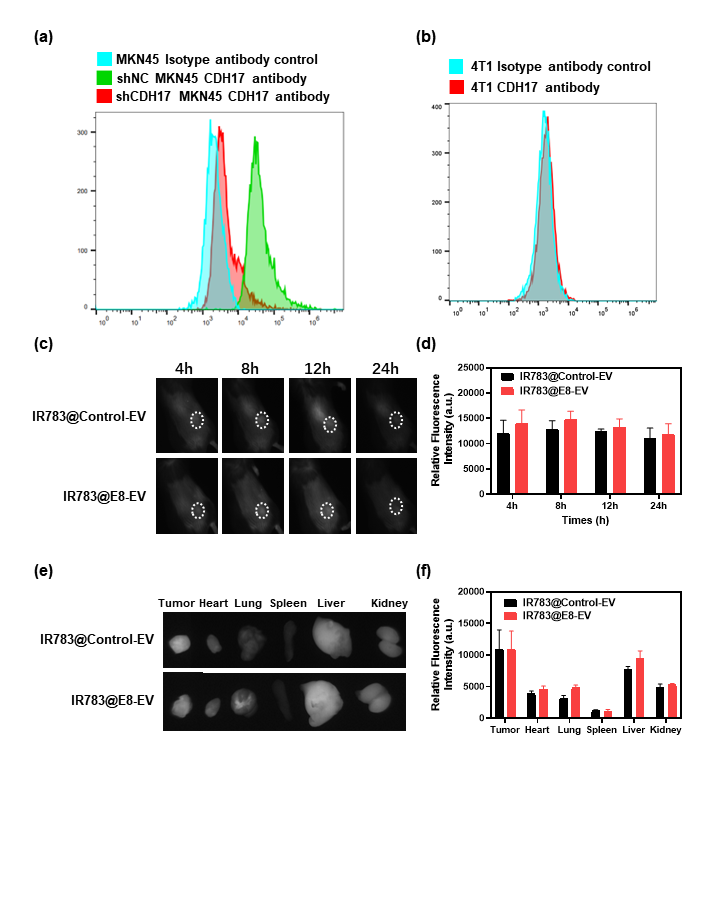


**Figure S5.** (a) CDH17 detection with flow cytometry in MKN 45 cells with/without CDH17 knockdown. (b) CDH17 detection with flow cytometry in 4T1 cells (c) In vivo NIR-II fluorescence images and (d) quantitation of the 4T1 tumors at 4, 8, 12, 24 h using IR783 as the imaging agent. (e) The fluorescence images and (f) the quantitation of main organs and tumors at 24 h postinjection, n = 3.


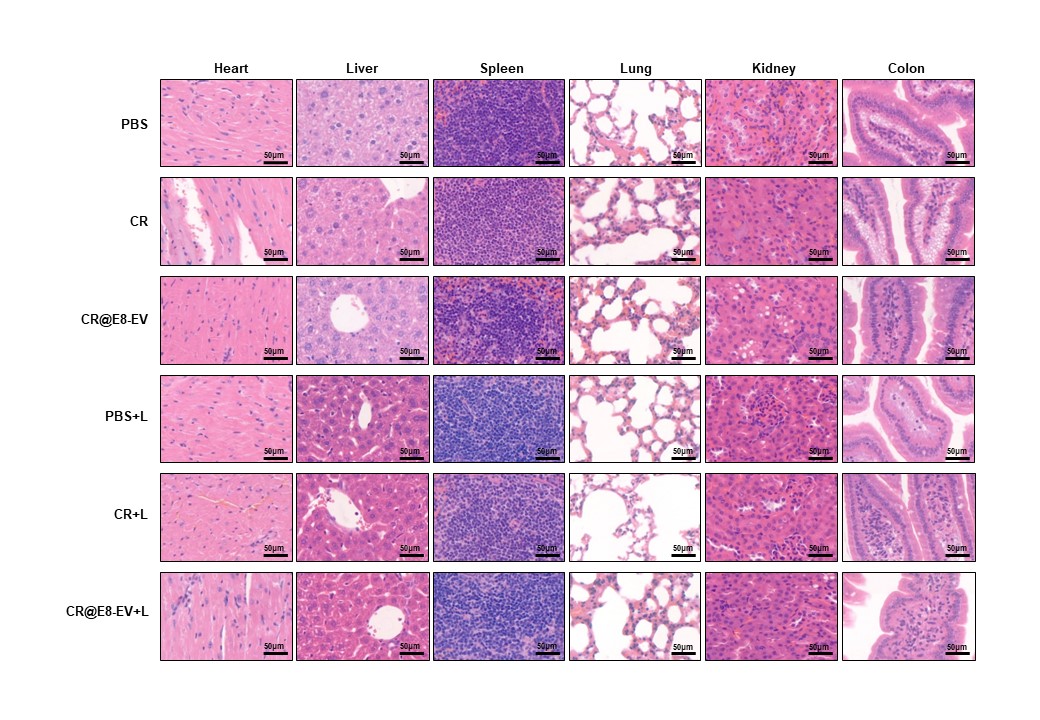


**Figure S6.** H&E analysis of the major organs at day 16 after different treatments.

**References**

1. Capodilupo AL, Vergaro V, Accorsi G, Fabiano E, Baldassarre F, Corrente GA, Gigli G, Ciccarella G. A series of diphenylamine-fluorenone derivatives as potential fluorescent probes for neuroblastoma cell staining. Tetrahedron 2016;72:2920–8.
2. Sun J, Zhao E, Liang J, Li H, Zhao S, Wang G, Gu X, Tang BZ. Diradical-featured organic small-molecule photothermal material with high-spin state in dimers for ultra-broadband solar energy harvesting. Adv Mater. 2022;34:e2108048.
